# Efficient Energy Saving Scenarios for Indoor PM_2.5_ Management in an Apartment of South Korea

**DOI:** 10.3390/toxics10100609

**Published:** 2022-10-13

**Authors:** Younghun Kim, Dongho Shin, Kee-Jung Hong, Gunhee Lee, Sang Bok Kim, Inyong Park, Bangwoo Han, Jungho Hwang

**Affiliations:** 1 Department of Sustainable Environment Research, Korea Institute of Machinery & Materials, Daejeon 34103, Korea; 2 Mechanical Engineering, Yonsei University, 50 Yonsei-ro, Seoul 03722, Korea

**Keywords:** PM_2.5_, air purifier, energy consumption, ventilation, indoor air quality

## Abstract

Indoor PM_2.5_ must be effectively controlled to minimize adverse impacts on public health. Cooking is one of the main sources of PM_2.5_ in residential areas, and indoor air quality (IAQ) management methods such as natural and mechanical ventilation, range hood, and air purifier are typically used to reduce cooking-generated PM_2.5_ concentrations. However, studies on the combined effects of various IAQ management methods on indoor PM_2.5_ reduction and energy consumption are limited. In this study, a theoretical model was established to estimate the performance of various IAQ management methods for controlling indoor PM_2.5_ concentrations and energy consumption. The model was verified by comparative experiments in which, various IAQ management methods were operated individually or combined. Seasonal energy consumption was calculated through the verified model, and energy consumption saving scenarios were derived for maintaining indoor PM_2.5_ concentrations less than 10 μg/m^3^, a World Health Organization annual guideline, under fair and poor outdoor PM_2.5_ concentrations of 15 and 50 μg/m^3^, respectively. Based on our results, we found that energy consumption could be reduced significantly by applying natural ventilation in spring, autumn, and summer and mechanical ventilation in winter. Our study identified efficient energy saving PM_2.5_ management scenarios using various IAQ management methods by predicting indoor PM_2.5_ concentration and energy consumption according to the annual life patterns of typical residents in South Korea.

## 1. Introduction

Most people spend 90% of their day indoors and thus can be easily exposed to indoor air pollutants [1]. PM_2.5_ is an indoor air pollutant and Group 1 carcinogen [2,3], and indoor PM_2.5_ has been found to exist at concentrations up to five times higher than that under outdoor conditions [4]. The indoor environment contains various harmful substances such as volatile organic compounds (VOCs), carbon dioxide (CO_2_), cigarette smoke, and allergens and is therefore one of the primary vectors of human respiratory disease [5,6,7]. Aerosols in indoor environments containing PM_2.5_ can promote the transmission of viruses such as SARS-CoV-2 [8,9], thus making management of indoor air quality (IAQ) crucial.

There are various indoor environments, including industrial, commercial, and residential facilities. The current methods for managing IAQ include natural ventilation, mechanical ventilation, air purifier use, and situation-specific ventilation, such as range hood use. Mechanical ventilation systems manage IAQ by supplying relatively clean outdoor air to the indoor environment and exhausting contaminated indoor air to the outdoors. Compared to natural ventilation, mechanical ventilation systems have the advantage of improving energy efficiency by utilizing an energy recovery heat exchanger. Therefore, mechanical ventilation has been actively studied to evaluate its effectiveness in particle and harmful gas reduction [10] and in promoting efficient energy consumption [11,12,13].

Many studies have assessed the influence of natural ventilation and range hoods on IAQ improvement. The effect of outdoor PM_2.5_ on indoor PM_2.5_ under natural ventilation conditions [4,14] and indoor particle deposition by natural ventilation [15] have also been investigated. Range hoods reduce concentrations of harmful substances generated by cooking, and related studies have assessed the efficacy of range hoods under different cooking conditions. Specifically, previous studies have assessed the combined impacts of natural ventilation and range hoods on indoor PM_2.5_ reduction [16] as well as the particle reduction characteristics of range hoods under various cooking conditions [17].

Air purifiers manage IAQ by removing PM_2.5_ and harmful gases from enclosed spaces. Previous studies have assessed the particle removal mechanisms of air purifiers using electrostatic precipitators, mechanical filters, and photocatalysts [18,19,20]. Studies have also investigated the performance of air purifiers under various indoor environmental conditions, contamination from different indoor PM_2.5_ sources [21,22,23], and varying outdoor PM_2.5_ concentrations [24].

Several studies have assessed the impacts of individual IAQ management methods; however, few have evaluated the combined effects of different IAQ management methods on indoor PM_2.5_ reduction and energy consumption. Moreover, many studies have assessed and determined the most efficient energy systems for indoor industrial and commercial facilities [25,26,27], but studies on the energy management for indoor air in residential houses are limited.

In this study, we proposed seasonal efficient energy consumption reduction scenarios for indoor PM_2.5_ management. A theoretical model for estimating indoor PM_2.5_ and energy consumption in residences was established to derive effective energy consumption scenarios. This theoretical model considered various IAQ management methods (such as mechanical ventilation, natural ventilation, range hood, and air purifier) with energy consumption considering the power consumption of each IAQ management method and enthalpy loss between indoor and outdoor air. To validate our theoretical model, the results were verified through comparison with experimental results for identical IAQ management conditions. Through our estimations, we derived seasonal energy consumption scenarios considering various IAQ management methods for maintaining average indoor PM_2.5_ concentrations less than 10 μg/m^3^, according to the World Health Organization’s (WHO) recommendation [28,29].

## 2. Materials and Methods

### 2.1. Experimental Design

**Figure 1:**
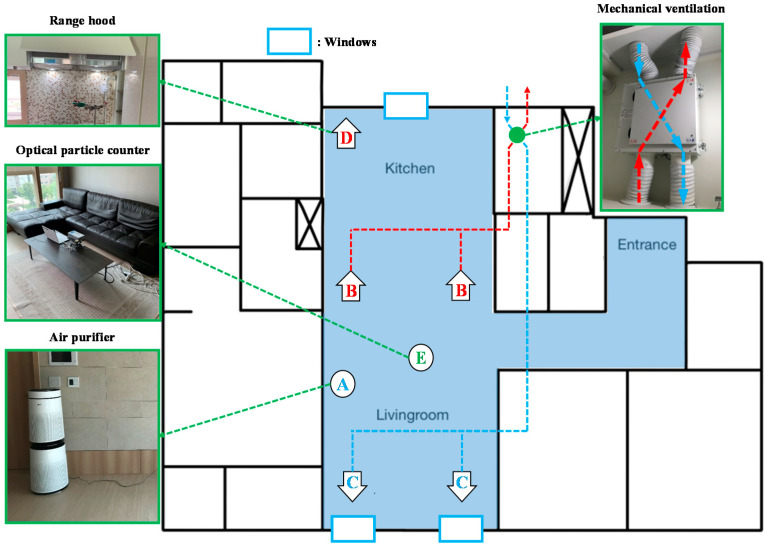
Apartment setup for experiments. Red letters and lines: IAQ management methods to exhaust indoor PM_2.5_. Blue letters and lines: IAQ management methods to supply outdoor PM_2.5_ or clean indoor PM_2.5_.

Figure 1 presents a schematic of the measuring equipment layout and IAQ management methods applied in the apartment used for this study. The apartment building was built in 2018, and the private area is approximately 72.5 m^2^. The air change rate (*ACH50*) of the apartment was approximately 2.3 h^−1^. The experiments only considered the kitchen and living room, which had a combined area of approximately 36.7 m^2^ and combined volume of approximately 84.6 m^3^. An air purifier was located in the living room (A, AS247DWE, LG Electronics, Seoul, Korea). Two supply and exhaust ports (B, C) for a mechanical ventilation system were installed on the ceiling (AP-0150CS, AP Co., Seoul, Korea). The range hood was located on the wall above the gas stove in the kitchen (D, HDH-90S, Haatz, Seoul, Korea). Particle concentration was measured between the kitchen and living room using a light scattering particle measuring device (E, Optical particle counter [OPC], Model 1.109, GRIMM Aerosol Technik Co., Ainring, Germany). Particle concentration were measured by OPC at 6 s intervals, and the average particle concentration data during 1 min were used. The windows were located in the living room and kitchen; the wind flowed through areas of 3.8 m^2^ (living room) and 0.23 m^2^ (kitchen). The wind speed through the window was measured by an anemometer (AMI310, KIMO Inst., Montpon, France) placed at the kitchen window. 

During the experiments, we measured cooking-generated PM_2.5_ concentration in the apartment. Approximately 100 g of food material (grilled roasted pork belly) was used for each cooking experiment. The IAQ management methods were initiated at the start of cooking, and indoor PM_2.5_ was reduced by their continuous operation until 20–30 min after cooking was completed.

### 2.2. Theoretical Model of Indoor PM2.5 and Energy Consumption

**Figure 2:**
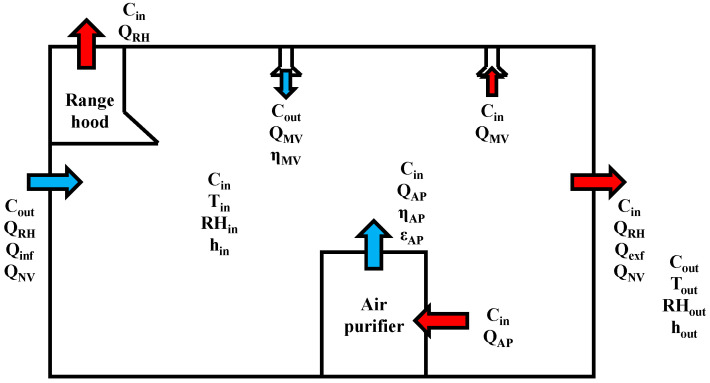
Schematic diagram of theoretical model for indoor PM_2.5_ and energy consumption. Red arrows: IAQ management methods to exhaust indoor PM_2.5_. Blue arrows: IAQ management methods to supply outdoor PM_2.5_ or clean indoor PM_2.5_.

Figure 2 is a schematic of the theoretical model parameters affecting indoor PM_2.5_ and indoor heat load in a residential environment. The theoretical model of indoor PM_2.5_ considers apartment specifications (e.g., infiltration, exfiltration, and deposition) as well as various configurations of IAQ management use and timing. The theoretical model of indoor PM_2.5_ is shown in Equation (1):

(1) $$V\frac{dC_{in}}{dt}=\left(1-\eta_{MV}\right)C_{out}Q_{MV}-C_{in}Q_{MV}+C_{out}Q_{RH}-C_{in}Q_{RH}+C_{out}Q_{NV}-C_{in}Q_{NV}-\eta_{AP}C_{in}Q_{AP}\varepsilon_{AP}-V\dot{S}C_{in}+C_{out}Q_{inf}-C_{in}Q_{exf}$$

The parameters of Equation (1) are detailed in Table 1. Equation (1) shows the indoor PM_2.5_ according to time and is an expression of the relationship between outdoor PM_2.5_ concentration, IAQ management methods, and apartment specifications (infiltration, exfiltration, and deposition). The flow rate of the mechanical ventilation system ($Q_{MV}$) was approximately 0.7 m^3^/min, and the ventilation rate through mechanical ventilation was 0.5 h^−1^, which satisfy requirements of approximately one third of countries [30]. The flow rate of the air purifier ($Q_{AP}$) was approximately 2.0, 5.0, 7.5, and 11.0 m^3^/min according to operation modes 1–4. The flow rate of the range hood ($Q_{RH}$) was approximately 3.1 m^3^/min. The natural ventilation flow rate ($Q_{NV}$) was calculated by the wind speed at the kitchen window, and the experiments and theoretical analysis were conducted under the condition that the living room and kitchen windows facing each other were both kept open for the set natural ventilation period. The mechanical ventilation system PM_2.5_ removal efficiency ($\eta_{MV}$) was approximately 70% (MERV13), and the air purifier PM_2.5_ removal efficiency ($\eta_{AP}$) was approximately 99.9% (HEPA). The short-circuiting factor ($\varepsilon_{AP}$) of the air purifier, which indicates the mixing characteristics of clean air with indoor air, was set to approximately 0.75 [24,31]. Infiltration and exfiltration ($Q_{inf}$ and $Q_{exf}$) were calculated using the leakage rate (*ACH*50) for the apartment under the condition that natural and mechanical ventilation methods and the range hood were inoperative. The leakage rate was measured under depressurized and pressurized conditions and was calculated using *V* × *ACH*50/20, similar to the actual condition [32,33]. The deposition rate ($\dot{S}$) of PM_2.5_ in the apartment was set to 0.0008/min [24,34].

We calculated the energy consumption of each IAQ management method by considering indoor and outdoor temperatures and humidity conditions:

(2) $$h_{in}=C_{P,\mathrm{dry}\;air}T_{in}\left(t\right)+x_{in}\left(t\right)\left(C_{P,vapor}T_{in}+h_{vapor}\right),$$

(3) $$h_{out}=C_{P,\mathrm{dry}\;air}T_{out}\left(t\right)+x_{out}\left(t\right)\left(C_{P,vapor}T_{out}+h_{vapor}\right),$$

(4) $$\Delta h=h_{out}-h_{in},$$

(5) $$H=\rho_{air}\Delta h\left(\varepsilon_{heat,MV}Q_{MV}+Q_{RH}+Q_{NV}\right)$$

(6) $$E_{T}=\left(H+E_{MV}+E_{RH}+E_{AP}\right)\times hour,$$

where $h_{in}$ is the indoor enthalpy, $h_{out}$ is the outdoor enthalpy, $T_{in}$ is the indoor temperature, $T_{out}$ is the outdoor temperature, $x_{in}$ is the indoor absolute humidity, $x_{out}$ is the outdoor absolute humidity, $C_{P,\mathrm{dry}\;air}$ is the specific heat capacity at constant pressure for dry air, $C_{P,vapor}$ is the specific heat capacity at constant pressure for water vapor, $h_{vapor}$ is water vaporization enthalpy, t is time, H is the heat load, $\varepsilon_{heat,MV}$ is the heat change efficiency for mechanical ventilation, $\rho_{air}$ is the air density, $E_{MV}$ is the power consumption of mechanical ventilation, $E_{RH}$ is the power consumption of the range hood, $E_{AP}$ is the power consumption of the air purifier, and $E_{T}$ is the total energy consumption.

We calculated indoor and outdoor enthalpy ($h_{in}$ and $h_{out}$) using indoor and outdoor temperature and humidity in Equations (2) and (3), which consider sensible and latent heat. The difference between indoor and outdoor enthalpy values (Δh) was obtained using Equation (4), and the heat energy loss (H) due to the supply of outdoor air via the IAQ management methods was derived using Equation (5). Mechanical ventilation generally applies an energy recovery system to account for heat exchange between exhausted indoor air and supplied outdoor air, which reduces heat energy loss. The heat change efficiency ($\varepsilon_{heat,MV}$) was assumed 0.5 for both heating and cooling. Mechanical ventilation systems, range hoods, and air purifiers consume electricity during operation. The power consumption of the mechanical ventilation system ($E_{MV}$) and range hood ($E_{RH}$) were approximately 63.1 and 98.0 W, respectively, and that of the air purifier ($E_{AP}$) was approximately 8.9, 16.3, 27.9, and 62.4 W for operation modes 1–4. Total energy consumption ($E_{T}$) was obtained by considering the heat load, power consumption, and device usage time, as shown in Equation (6), over 16 h (7:00 to 23:00). This period was selected as it is representative of the time of day during which residents spend most of their time in the living room and kitchen.

Table 2 presents the indoor and outdoor temperature and humidity every hour during each season for the calculation of energy consumption. We used hourly data of outdoor temperature and humidity in Seoul, Korea, in 2019 provided by the Korea Meteorological Administration (KMA) [35]. We used the average temperature and humidity data in March–May for spring, September–November for autumn, June–August for summer, and December–February for winter. Indoor temperature and humidity were calculated in a prior study, and the deviations for each season were ±1.8 °C and ±9.5% in spring/autumn, ±2 °C and ±9.7% in summer, and ±1.6 °C and ±9.5% in winter [36]. The set temperature and humidity of typical living environments were assumed to be the indoor temperature and humidity in each season. The indoor and outdoor enthalpy (to calculate the energy consumption) was calculated using the indoor and outdoor temperature and humidity for each season, which was then used to calculate the energy consumption according to the IAQ management methods. We assumed that the difference in enthalpy within the deviation of the set temperature was outside the range of the residents’ perception and was therefore ignored.

Furthermore, we derived the most efficient scenario to reduce energy consumption based on total energy consumption per season at which indoor PM_2.5_ concentration below 10 μg/m^3^ is maintained, according to the WHO recommendation.

## 3. Results and Discussion

**Figure 3:**
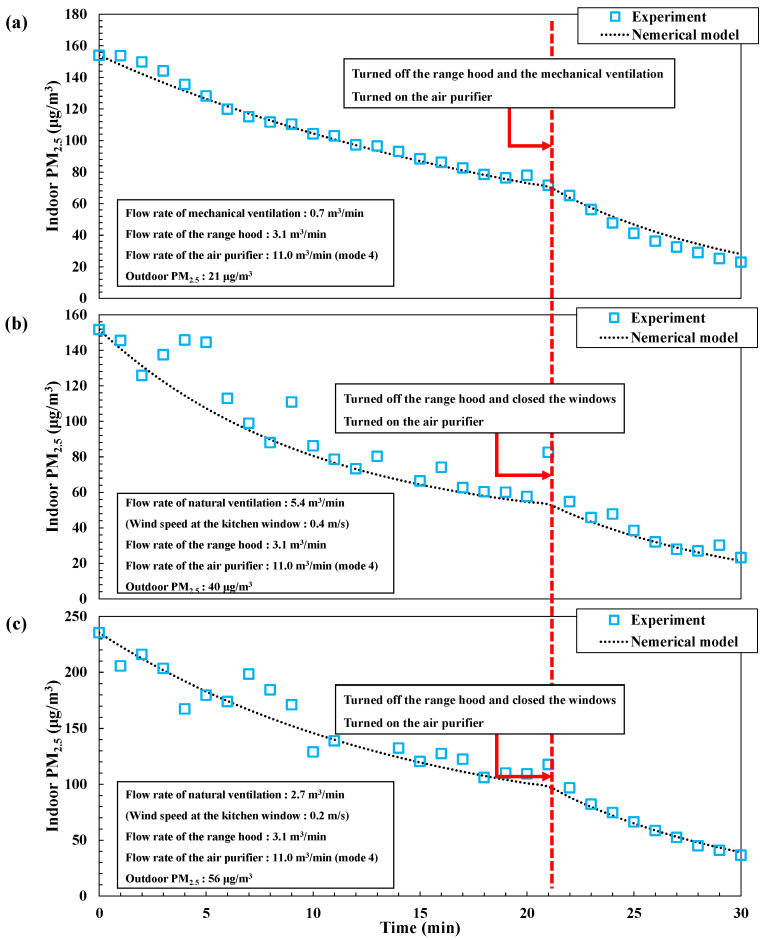
Comparison of indoor PM_2.5_ concentrations in experiments and theoretical model. (**a**) Mechanical ventilation + range hood + air purifier. (**b**) Natural ventilation (0.4 m/s) + range hood + air purifier. (**c**) Natural ventilation (0.2 m/s) + range hood + air purifier.

We compared theoretical and experimental cooking-generated PM_2.5_ results for each IAQ management method to validate the theoretical solution derived by Equation (1); the results are shown in Figure 3. We obtained the following results for each IAQ configuration: mechanical ventilation + range hood were applied for 20 min, and then the air purifier was operated in mode 4 for 10 min; the wind speed at the kitchen window was 0.2 m/s and outdoor PM_2.5_ was 21 μg/m^3^ (Figure 3a). Natural ventilation + range hood use were applied for 20 min, and then the air purifier was operated in mode 4 for 10 min (Figure 3b,c). For natural ventilation + range hood use, the wind speed at the kitchen window was evaluated at 0.4 m/s and 0.2 m/s and outdoor PM_2.5_ was 40 and 56 μg/m^3^ (Figure 3b and 3c, respectively). For the conditions described in Figure 3b,c, the initial indoor PM_2.5_ was approximately 150 μg/m^3^. Figure 3b shows that although outdoor PM_2.5_ was 40 μg/m^3^, which is twice as high as that in Figure 3a, indoor PM_2.5_ decreased to 57 μg/m^3^ lower than 77 μg/m^3^ in Figure 3a after 20 min due to high flow rate of natural ventilation. The results of the theoretical model were significantly consistent with the experimental results. The theoretical model results show similar reduction characteristics to the experimental results under the same IAQ management conditions with different outdoor conditions. Therefore, our proposed theoretical model was deemed suitable to estimate indoor PM_2.5_ concentrations by the operations of IAQ management systems.

**Figure 4:**
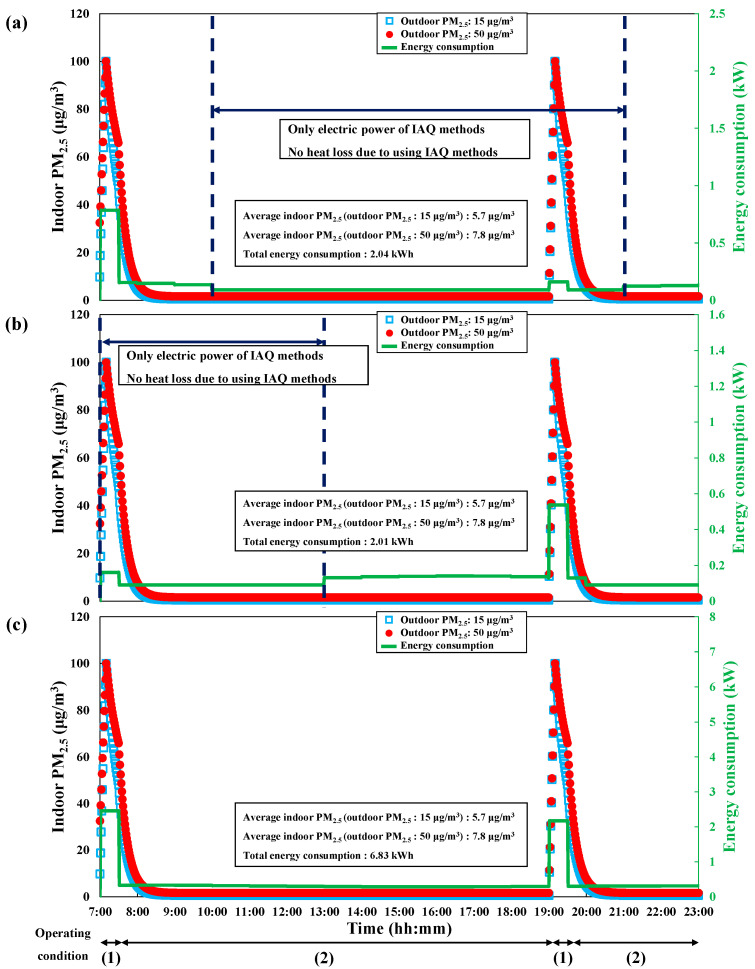
Changes in PM_2.5_ concentration and energy consumption with time to maintain average PM_2.5_ < 10 μg/m^3^ under general energy consumption scenario. (**a**) Spring/autumn. (**b**) Summer. (**c**) Winter.

We simulated the indoor PM_2.5_ concentration and energy consumption for each season based on twice-daily cooking, as cooking is the main cause of indoor PM_2.5_ in residential environments. The general energy consumption scenario was defined based on the combined use of the range hood, mechanical ventilation, and air purifier for IAQ management, regardless of season. Figure 4 shows the general energy consumption scenario for each season under the condition that the average indoor PM_2.5_ concentration was maintained below 10 μg/m^3^ when the outdoor PM_2.5_ concentration was 15 and 50 μg/m^3^. For the experiment, cooking occurred twice daily at 7:00 and 19:00. The initial indoor PM_2.5_ concentration at 7:00 (beginning of the scenario) was set to approximately 0.65 of the outdoor PM_2.5_ concentration, according to the I/O ratio [37,38]. The cooking-generated PM_2.5_ was assumed to increase linearly to 100 μg/m^3^ for 10 min in all situations. In addition, we used the same ventilation conditions of 0.5 h^−1^ in the natural and mechanical ventilation scenarios to accurately compare their energy consumption. Natural ventilation was set to be performed for 30 min thrice a day per situation, with a flow rate set to approximately 7.5 m^3^/min. The range hood was set to be operated as soon as the cooking started and continuously operated for 30 min (10 min during cooking and 20 min after cooking). After 20 min from the end of cooking, the air purifier was also set to operate in all conditions. The operating conditions of the IAQ management methods are shown in Table 3. Table 4 shows the timetable applied for each operating condition of the general energy consumption scenario. Moreover, the section of the operating conditions is shown at the bottom of each graph in Figure 4.

For each season, the mechanical ventilation and the range hood (Operating condition 1) were set to be operated at 7:00–7:30 and 19:00–19:30, which corresponded to 10 min during cooking and 20 min after cooking. The mechanical ventilation and air purifier (Operating condition 2) were set to be operated at 07:30–19:00 and 19:30–23:00. The air purifier was set to mode 3 (clean air delivery rate (CADR) = 7.0 m^3^/min), which is close to the CADR (84.6 m^3^ × 4.8 h^−1^ / 60 min = 6.8 m^3^/min) suggested by the standard of the Association of Home Appliance Manufacturers (AHAM) [39] and the United States Environmental Protection Agency (EPA) [40,41].

In the general energy consumption scenario for spring/autumn, we found that the use of the range hood and mechanical ventilation caused significant heat loss at 7:00–10:00, when the indoor and outdoor enthalpy difference was the largest. In contrast, we observed less heat loss after 10:00 due to the increase in outdoor temperature, and the electric power of each method was dominantly consumed among the total energy consumption. We observed less heat loss during the application of each method in summer because of the relatively low outdoor temperature at 7:00–13:00; however, due to the increase in outdoor temperature, we observed an increase in heat gain from 13:00 when applying the mechanical ventilation, which reduced again after 20:00. Unlike the other seasons, heat loss occurred throughout all hours in winter, as the outdoor temperature was significantly lower than the indoor temperature throughout the day. This caused a large difference between indoor and outdoor enthalpy. Significant heat loss still occurred even when using the mechanical ventilation energy recovery system.

When the outdoor PM_2.5_ concentration was set at 15 μg/m^3^, the indoor average PM_2.5_ concentration in all seasons was 5.7 μg/m^3^, and the energy consumption by the IAQ management methods was approximately 2.04, 2.01, and 6.83 kWh in spring/autumn, summer, and winter, respectively. When the outdoor PM_2.5_ concentration was set at 50 μg/m^3^, the indoor average PM_2.5_ concentration was 7.8 μg/m^3^ in all seasons, and the energy consumption by the IAQ management methods was equal to the consumption under an outdoor PM_2.5_ concentration of 15 μg/m^3^.

**Figure 5:**
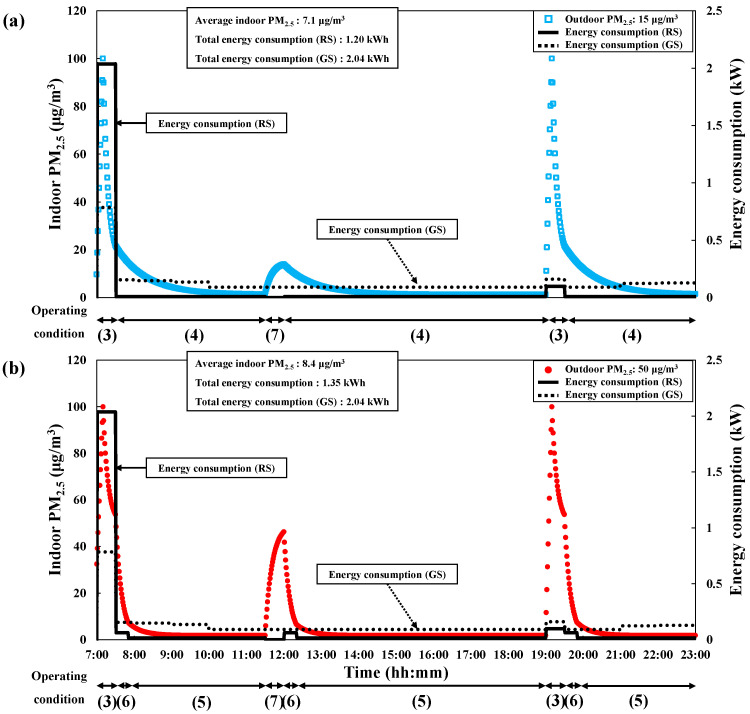
Changes in PM_2.5_ concentration and energy consumption with time to maintain average PM_2.5_ < 10 μg/m^3^ under the energy consumption reduction scenario in spring/autumn (GS: general scenario, RS: reduction scenario). (**a**) Outdoor PM_2.5_: 15 μg/m^3^. (**b**) Outdoor PM_2.5_: 50 μg/m^3^.

**Figure 6:**
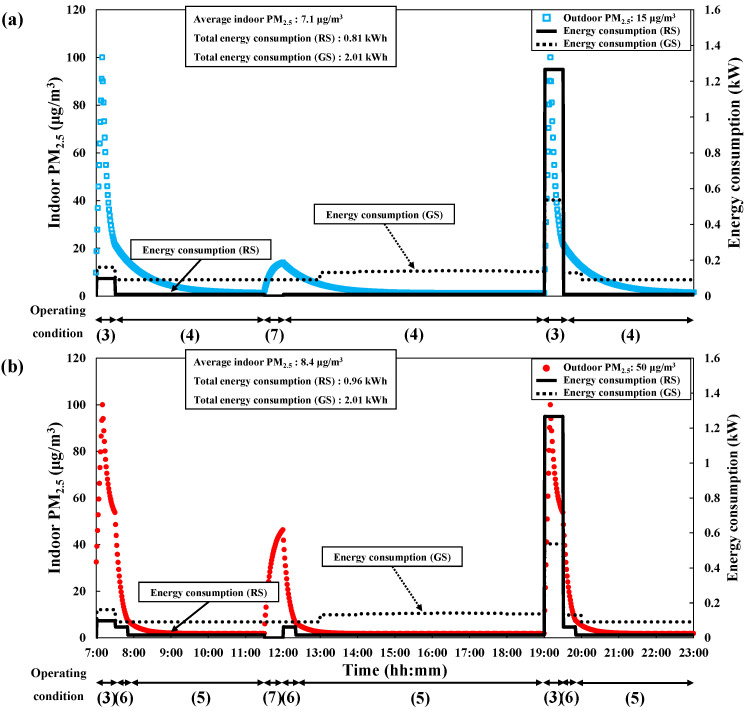
Changes in PM_2.5_ concentration and energy consumption with time to maintain average PM_2.5_ < 10 μg/m^3^ under the energy consumption reduction scenario in summer (GS: general scenario, RS: reduction scenario). (**a**) Outdoor PM_2.5_: 15 μg/m^3^. (**b**) Outdoor PM_2.5_: 50 μg/m^3^.

Figure 5 and Figure 6 shows the energy consumption (by IAQ management method) and indoor PM_2.5_ concentration with time for the energy consumption reduction scenario in spring/ autumn and summer, respectively. Results were obtained under the assumptions that outdoor PM_2.5_ was 15 μg/m^3^ (Figure 5a and Figure 6a) or 50 μg/m^3^ (Figure 5b and Figure 6b). Table 5 shows the timetable of operating conditions for IAQ management methods under each energy consumption reduction scenario. In the general energy consumption scenario for spring/autumn and summer, we found that electric power was dominantly consumed by mechanical ventilation and the air purifier, as there was little difference between the indoor and outdoor enthalpy during 8:00–20:00 in spring/autumn and 7:00–12:00 in summer. Thus, natural ventilation instead of mechanical ventilation can be effectively used to reduce energy consumption in the spring, autumn, and summer. The operating conditions of IAQ management methods with time considering an outdoor PM_2.5_ of 15 μg/m^3^ in spring/autumn and summer were as follows: during 7:00–07:30 and 19:00–19:30 (cooking time), the range hood and natural ventilation were set to be applied (Operating condition 3) and additional natural ventilation was set to be applied during 11:30–12:00 (Operating condition 7) to satisfy the daily ventilation requirement (average ventilation rate 0.5 h^−1^). During 07:00–11:30, 12:00–19:00, and 19:30–23:00, the air purifier was set to be operated in mode 1 (Operating condition 4) instead of mode 3 to reduce its power consumption. 

Considering outdoor PM_2.5_ concentrations of 50 μg/m^3^ in spring/autumn and summer, the same operating conditions were set as those for an outdoor PM_2.5_ concentration of 15 μg/m^3^, except for the operation conditions of the air purifier. During 7:00–07:30 and 19:00–19:30 (cooking time), the indoor PM_2.5_ concentration converged to approximately 50 μg/m^3^, which matched the outdoor PM_2.5_ concentration, despite using the range hood and natural ventilation for 30 min. During 7:30–8:00, 12:00–12:30, and 19:30–20:00 (after cooking and ventilation), the air purifier was set to be operated in mode 4 (i.e., the maximum mode; Operating condition 6) to rapidly reduce indoor PM_2.5_ concentrations. During 8:00–11:30 and 20:00–23:00, the air purifier mode was set to mode 2 (Operating condition 5) to maintain a daily average indoor PM_2.5_ concentration <10 μg/m^3^. Moreover, even after only natural ventilation was applied in the daytime (at 11:30–12:00), the air purifier operating mode was set to mode 4 (Operating condition 6) at 12:00–12:30 to rapidly reduce indoor PM_2.5_, which had increased due to the supply of outdoor air during ventilation. By applying natural ventilation instead of mechanical ventilation, heat loss could be increased due to the supply of outdoor air without heat exchange during 7:00–7:30 in spring/autumn and 19:00–19:30 in summer. However, electric power was not consumed in natural ventilation, thus yielding reduced total energy consumption.

**Figure 7:**
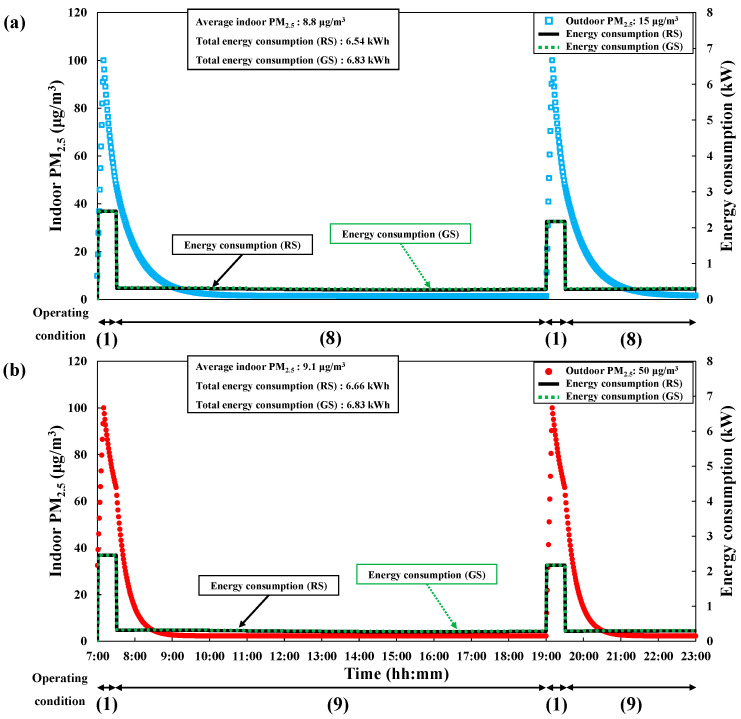
Changes in PM_2.5_ concentration and energy consumption with time to maintain average PM_2.5_ < 10 μg/m^3^ under the energy consumption reduction scenario in winter (GS: general scenario, RS: reduction scenario). (**a**) Outdoor PM_2.5_: 15 μg/m^3^. (**b**) Outdoor PM_2.5_: 50 μg/m^3^.

The energy consumption reduction scenario in winter is shown in Figure 7. Results were obtained assuming that the outdoor PM_2.5_ concentrations were 15 or 50 μg/m^3^ (Figure 7a and 7b, respectively). The timetable of the operating conditions for the energy consumption reduction scenario in winter is shown in Table 6. In contrast to its utility in other seasons, natural ventilation could not reduce energy consumption in winter due to the continual loss of heat. However, energy consumption could be reduced while satisfying the indoor PM_2.5_ concentration by applying mechanical ventilation (equipped with an energy recovery system) as well as optimizing the operation mode of the air purifier. During 07:00–07:30 in winter, mechanical ventilation and the range hood were set to be applied (Operating condition 1), while the mechanical ventilation and the air purifier (Operating conditions 8 and 9) were set to be applied at other times (07:30–19:00 and 19:30–23:00). The operating condition of the air purifier was set to modes 1 and 2 when the outdoor PM_2.5_ concentrations were 15 and 50 μg/m^3^, respectively.

**Figure 8:**
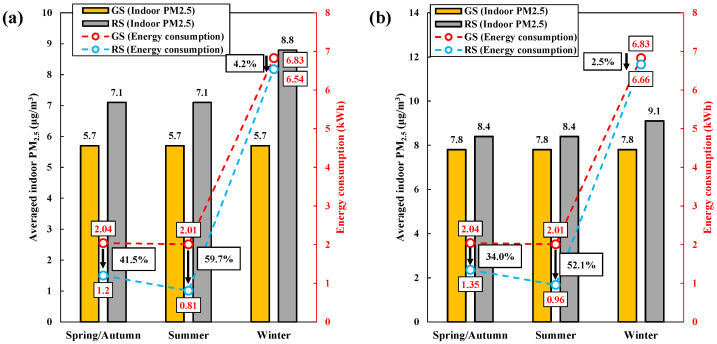
Averaged indoor PM_2.5_ and energy consumption in general energy consumption and energy consumption reduction scenarios (GS: general scenario, RS: reduction scenario). (**a**) Outdoor PM_2.5_: 15 μg/m^3^. (**b**) Outdoor PM_2.5_: 50 μg/m^3^.

Figure 8 shows the average indoor PM_2.5_, energy consumption, and energy savings (reduction rate) for the energy consumption reduction scenario compared to the general energy consumption scenario. In the energy consumption reduction scenario at outdoor PM_2.5_ concentrations of 15 μg/m^3^, the average indoor PM_2.5_ concentrations for spring/autumn, summer, and winter were approximately 7.1, 7.1, and 8.8 μg/m^3^, respectively. The corresponding energy consumption from the use of the IAQ management methods was 1.20, 0.81, and 6.54 kWh, respectively. Compared to those in the general scenario, the corresponding energy savings (reduction rate) through the reduction scenario were approximately 0.85 (41.5%), 1.20 (59.7%), and 0.28 (4.2%) kWh, respectively. When the outdoor PM_2.5_ concentration was 50 μg/m^3^, the indoor PM_2.5_ concentrations were 8.4, 8.4, and 9.1 μg/m^3^; the energy consumption levels were 1.35, 0.96, and 6.66 kWh; and the energy savings (reduction rates) were approximately 0.69 (34.0%), 1.05 (52.1%), and 0.17 (2.5%) kWh, respectively, compared to those in the general scenario. The relatively high outdoor PM_2.5_ concentration of 50 μg/m^3^ resulted in a slight increase in energy consumption (0.12–0.15 kWh) to effectively manage indoor PM_2.5_ concentrations compared to the required energy consumption at an outdoor PM_2.5_ concentration of 15 μg/m^3^.

**Figure 9:**
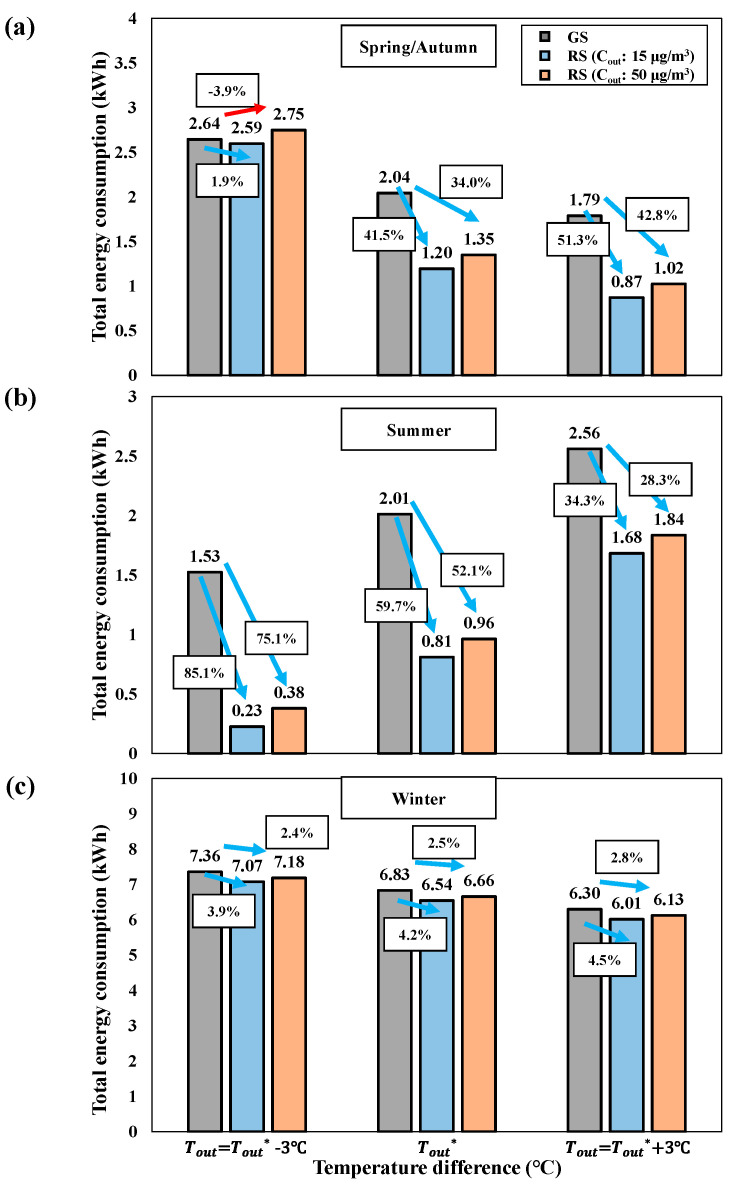
Total energy consumption of scenarios according to outdoor temperature in each season (GS: general scenario, RS: reduction scenario). (**a**) Spring/Autumn (**b**) Summer. (**c**) Winter.

In order to further analyze our scenario model, we compared the energy consumption when the outdoor temperature was changed in each season’s temperature and humidity conditions (Table 4). Figure 9 shows the energy consumption of each scenario with a difference of ±3 °C in the outdoor temperature ($T_{out}{}^{*}$) under the existing temperature and humidity conditions (Table 4) for each season. It shows that the reduction scenario through our scenario model is effective in terms of energy consumption under the outdoor temperature conditions of all seasons except for the $T_{out}=T_{out}{}^{*}-3\;℃$ of spring/autumn. In the case of the reduction scenario at the $T_{out}=T_{out}{}^{*}-3\;℃$ with an outdoor PM_2.5_ of 15 μg/m^3^ in spring/autumn, although heat loss by applying natural ventilation increased than the $T_{out}{}^{*}$, the total energy consumption decreased compared to the general scenario because there was no power consumption for mechanical ventilation. However, In the case of reduction scenario at the $T_{out}=T_{out}{}^{*}-3\;℃$ temperature difference with an outdoor PM_2.5_ of 50 μg/m^3^ in spring/autumn, although power consumption of mechanical ventilation was not occurred, the total energy consumption increased compared to the general scenario due to the relatively high flow rate of the air purifier to maintain indoor PM_2.5_ below 10 μg/m^3^. In order to reduce the average energy consumption in spring/autumn, it is efficient to apply natural ventilation instead of mechanical ventilation, but in the case of high outdoor PM_2.5_ in early spring or late autumn when the outdoor temperature is low, heat loss due to natural ventilation and power consumption of the air purifier increase, therefore, applying mechanical ventilation is more effective for energy saving.

## 4. Conclusions

In this study, we established a theoretical model for evaluating indoor PM_2.5_ management and energy consumption in residential apartments in Korea. Our theoretical model of indoor PM_2.5_ considered various IAQ management methods, namely, natural ventilation, mechanical ventilation, range hood use, and air purifier use in relation to outdoor PM_2.5_ concentrations within the constraints of apartment specifications (i.e., infiltration, exfiltration, and deposition). The theoretical model for indoor PM_2.5_ estimation was verified by comparison with experimental results under the same conditions. Through the validated model, we derived energy consumption reduction scenarios using various IAQ management methods by season. Difference in enthalpy due to indoor and outdoor temperature and humidity was a major role in effectively achieving a target indoor PM_2.5_ concentration less than 10 μg/m^3^ while minimizing energy consumption. During the daytime in spring/autumn and summer, the role of the energy recovery system in the mechanical ventilation system was less effective compared to natural ventilation. However, in winter, which showed the highest difference between indoor and outdoor temperature and humidity, mechanical ventilation effectively reduced heat loss due to the difference in indoor and outdoor enthalpy. Depending on the season and outdoor PM_2.5_, energy consumption was reduced by 2.7–59.7% by introducing the appropriate energy consumption reduction scenario considering indoor and outdoor conditions.

This study proposes an efficient scenario-based IAQ management operation approach for each season that can effectively reduce energy consumption while maintaining indoor PM_2.5_ concentrations under 10 μg/m^3^. The scenarios proposed in this study are expected to provide useful guidelines for effective indoor PM_2.5_ management in residential facilities and homes. To further improve IAQ and minimize energy consumption in residential environments, future studies should incorporate real-time indoor and outdoor temperature and humidity data as well as PM_2.5_ concentrations under actual residential conditions.

## Figures and Tables

**Table 1:** Parameters of the theoretical model.

IAQ Management Method	Mechanical Ventilation	Natural Ventilation	Air Purifier	Range Hood
Flow rate (m^3^/min)	0.7 $\left(Q_{MV}\right)$	Wind speed × Kitchen window area $(Q_{NV}$)	2.0 (mode 1) 5.0 (mode 2) 7.5 (mode 3) 11.0 (mode 4) $(Q_{AP}$)	3.1 $(Q_{RH}$)
PM_2.5_ removal efficiency (%)	70 $(\eta_{MV}$)	-	99.9 $(\eta_{AP}$)	-
Short circuiting factor (-)	-	-	0.75 $(\varepsilon_{AP}$)	-
Volume of apartment (V, m^3^): 84.6 Deposition rate of apartment ($\dot{S}$, min^−1^): 0.0008 $\mathrm{Infiltration}\;\mathrm{and}\;\mathrm{exfiltration}\;\mathrm{of}\;\mathrm{apartment}\;(Q_{inf}$$\;\mathrm{and}\;Q_{exf}$, m^3^/min): 0.16 $\mathrm{Outdoor}\;{\mathrm{PM}}_{2.5}:\;C_{out}$ (μg/m^3^) $\mathrm{Indoor}\;{\mathrm{PM}}_{2.5}:\;C_{in}$ (μg/m^3^) Time: t (min)				

**Table 2:** Hourly indoor and outdoor temperature and humidity data by season.

Time (hh:mm)	Spring/Autumn				Summer				Winter			
	Outdoor		Indoor		Outdoor		Indoor		Outdoor		Indoor	
	$T_{out}\;({}^{\circ}C)$	RH (%)	$T_{in}\;({}^{\circ}C)$	RH (%)	$T_{out}\;({}^{\circ}C)$	RH (%)	$T_{in}\;({}^{\circ}C)$	RH (%)	$T_{out}\;({}^{\circ}C)$	RH (%)	$T_{in}\;({}^{\circ}C)$	RH (%)
07:00	13.7	70.0	24.6	31.5	22.3	76.5	26.6	51.1	−2.5	60.0	23.9	25.0
08:00	14.9	64.7	24.7	31.3	23.3	72.0	26.7	50.9	−2.6	59.8	23.9	25.0
09:00	16.6	58.2	24.8	31.2	24.4	67.6	26.8	50.7	−1.9	55.0	23.9	24.9
10:00	18.4	52.9	24.8	31.0	25.6	62.6	26.8	50.5	−0.3	49.8	24.0	24.8
11:00	19.8	48.4	24.9	30.9	26.6	59.0	26.9	50.3	1.2	45.2	24.1	24.6
12:00	20.9	45.3	25.0	30.8	27.4	56.6	26.9	50.2	2.5	41.5	24.2	24.5
13:00	21.7	42.8	25.0	30.7	28.1	54.1	27.0	50.0	3.4	39.4	24.3	24.4
14:00	22.1	41.8	25.0	30.7	28.7	52.5	27.0	49.9	4.1	37.9	24.3	24.4
15:00	22.3	42.1	25.0	30.7	29.1	51.2	27.0	49.9	4.5	37.4	24.4	24.4
16:00	22.1	42.7	25.0	30.7	29.0	52.3	27.0	50.0	4.3	37.6	24.4	24.4
17:00	21.4	46.2	25.0	30.9	28.6	54.3	27.0	50.1	3.6	40.1	24.3	24.5
18:00	20.3	49.8	24.9	31.0	27.8	56.8	27.0	50.3	2.4	44.0	24.2	24.6
19:00	19.0	53.4	24.9	31.1	26.8	60.5	26.9	50.5	1.7	47.3	24.2	24.7
20:00	18.1	56.5	24.8	31.2	25.7	64.6	26.8	50.7	1.0	49.9	24.1	24.7
21:00	17.4	58.6	24.8	31.2	25.0	67.3	26.8	50.8	0.6	51.7	24.1	24.8
22:00	16.8	60.5	24.8	31.3	24.4	69.5	26.8	50.9	0.2	53.2	24.1	24.8
23:00	16.2	62.4	24.7	31.3	23.9	71.0	26.7	51.0	−0.2	54.2	24.0	24.9

**Table 3:** Operating conditions of IAQ management methods (NV: natural ventilation, MV: mechanical ventilation, RH: range hood, and AP: air purifier).

Operating condition 1	MV + RH
Operating condition 2	MV + AP mode 3
Operating condition 3	NV + RH
Operating condition 4	AP mode 1
Operating condition 5	AP mode 2
Operating condition 6	AP mode 4
Operating condition 7	NV
Operating condition 8	MV + AP mode 1
Operating condition 9	MV + AP mode 2

**Table 4:** Timetable of IAQ management operating conditions for the general energy consumption scenario.

Time (hh:mm)	Operating Condition for Spring/Autumn, Summer, and Winter	Remark
07:00–07:30	1	Cooking (07:00–07:10)
07:30–19:00	2	-
19:00–19:30	1	Cooking (07:00–07:10)
19:30–23:00	2	-

**Table 5:** Timetable of IAQ management operating conditions for the energy consumption reduction scenario in spring/autumn and summer (NV: natural ventilation, MV: mechanical ventilation, RH: range hood, and AP: air purifier).

Time (hh:mm)	Operating Conditions for Spring/Autumn, and Summer		Remark
	Outdoor PM_2.5_: 15 μg/m^3^	Outdoor PM_2.5_: 50 μg/m^3^	
07:00–07:30	3 (NV + RH)	3 (NV + RH)	Cooking (07:00–07:10)
07:30–08:00	4 (AP mode 1)	6 (AP mode 4)	-
08:00–11:30	4 (AP mode 1)	5 (AP mode 2)	-
11:30–12:00	7 (NV)	7 (NV)	-
12:00–12:30	4 (AP mode 1)	6 (AP mode 4)	-
12:30–19:00	4 (AP mode 1)	5 (AP mode 2)	-
19:00–19:30	3 (NV + RH)	3 (NV + RH)	Cooking (07:00–07:10)
19:30–20:00	4 (AP mode 1)	6 (AP mode 4)	-
20:00–23:00	4 (AP mode 1)	5 (AP mode 2)	-

**Table 6:** Timetable of IAQ management operating conditions for the energy consumption reduction scenario in winter (NV: natural ventilation, MV: mechanical ventilation, RH: range hood, and AP: air purifier).

Time (hh:mm)	Operating Conditions for Winter		Remark
	Outdoor PM_2.5_: 15 μg/m^3^	Outdoor PM_2.5_: 50 μg/m^3^	
07:00–07:30	1 (MV + RH)	1 (MV + RH)	Cooking (07:00–07:10)
07:30–19:00	8 (MV + AP mode 1)	9 (MV + AP mode 2)	-
19:00–19:30	1 (MV + RH)	1 (MV + RH)	Cooking (07:00–07:10)
19:30–23:00	8 (MV + AP mode 1)	9 (MV + AP mode 2)	-

## Data Availability

Not applicable.
